# Perspectives: SARS-CoV-2 Spike Convergent Evolution as a Guide to Explore Adaptive Advantage

**DOI:** 10.3389/fcimb.2022.748948

**Published:** 2022-05-27

**Authors:** Jiri Zahradník, Jaroslav Nunvar, Gideon Schreiber

**Affiliations:** ^1^ Department of Biomolecular Sciences, Weizmann Institute of Science, Rehovot, Israel; ^2^ Department of Genetics and Microbiology, Faculty of Science, Charles University, Prague, Czechia; ^3^ Biotechnology and Biomedicine Centre of the Academy of Sciences and Charles University (BIOCEV), Vestec, Czechia

**Keywords:** SARS-CoV-2, convergent evolution, mutations, virus, spike (S) protein

## Abstract

Viruses rapidly co-evolve with their hosts. The 9 million sequenced SARS-CoV-2 genomes by March 2022 provide a detailed account of viral evolution, showing that all amino acids have been mutated many times. However, only a few became prominent in the viral population. Here, we investigated the emergence of the same mutations in unrelated parallel lineages and the extent of such convergent evolution on the molecular level in the spike (S) protein. We found that during the first phase of the pandemic (until mid 2021, before mass vaccination) 31 mutations evolved independently ≥3-times within separated lineages. These included all the key mutations in SARS-CoV-2 variants of concern (VOC) at that time, indicating their fundamental adaptive advantage. The omicron added many more mutations not frequently seen before, which can be attributed to the synergistic nature of these mutations, which is more difficult to evolve. The great majority (24/31) of S-protein mutations under convergent evolution tightly cluster in three functional domains; N-terminal domain, receptor-binding domain, and Furin cleavage site. Furthermore, among the S-protein receptor-binding motif mutations, ACE2 affinity-improving substitutions are favoured. Next, we determined the mutation space in the S protein that has been covered by SARS-CoV-2. We found that all amino acids that are reachable by single nucleotide changes have been probed multiple times in early 2021. The substitutions requiring two nucleotide changes have recently (late 2021) gained momentum and their numbers are increasing rapidly. These provide a large mutation landscape for SARS-CoV-2 future evolution, on which research should focus now.

## Results and Discussion

### Early Stages of SARS-CoV-2 Evolution

In the initial stages of the SARS-CoV-2 pandemic, virus evolution was shaped by selection imposed by a naïve host and the environment, resulting in new variants with adaptive advantage rapidly taking over previous strains. At this stage, global attention was focused on a number of major variants of concern: alpha (B.1.1.7), initially prominent in the United Kingdom, beta (B.1.351), discovered in South Africa, gamma (P.1) which has spread rapidly in the State of Amazonas and delta (B.1.617.2) spreading from India. With the widespread introduction of vaccines during the first half of 2021, SARS-CoV-2 evolution shifted towards immune evading variants, most notable omicron BA1 discovered in South Africa and most recently omicron BA2. Independent acquisitions of S-protein substitutions L452R, E484K/Q, N501Y, and Q677H in these and other lineages were analyzed in great detail (Cherian et al., 2021; Hodcroft et al., 2021; Lemmermann et al., 2021; Martin et al., 2021; Zhou et al., 2021). The intra-host SARS-CoV-2 genomic diversity (Ramazzotti et al., 2021) and the viral evolution in immunocompromised patients (Choi et al., 2020; Clark et al., 2021; Tarhini et al., 2021; Truong et al., 2021) also received a lot of attention. Yet studies focused on convergent (syn. parallel) evolution highlighting new potential mutations of interest and their combination are scarce. Convergent evolution is evidence that natural selection plays a pivotal role in the emergence of remarkable patterns of independently evolving mutations that are arising independently within the members of different lineages (Martin et al., 2021). Here, we analyzed the convergent evolution of SARS-CoV-2 spike protein (S-protein) amino acid (AA) changes which have emerged independently since early 2020 in three or more prominent lineages. In addition, an exhaustive analysis of all possible S-protein receptor-binding motif substitutions, which are reachable by single- and double-nucleotide mutations, was conducted with respect to their global presence and binding effect. Our findings map the peculiarities of the SARS-CoV-2 mutational landscape and reinforce the need of careful monitoring of SARS-CoV-2 evolution.

### Mutations With Putative Fitness Advantage Accumulate Independently in Multiple SARS-CoV-2 Lineages

**Figure 1:**
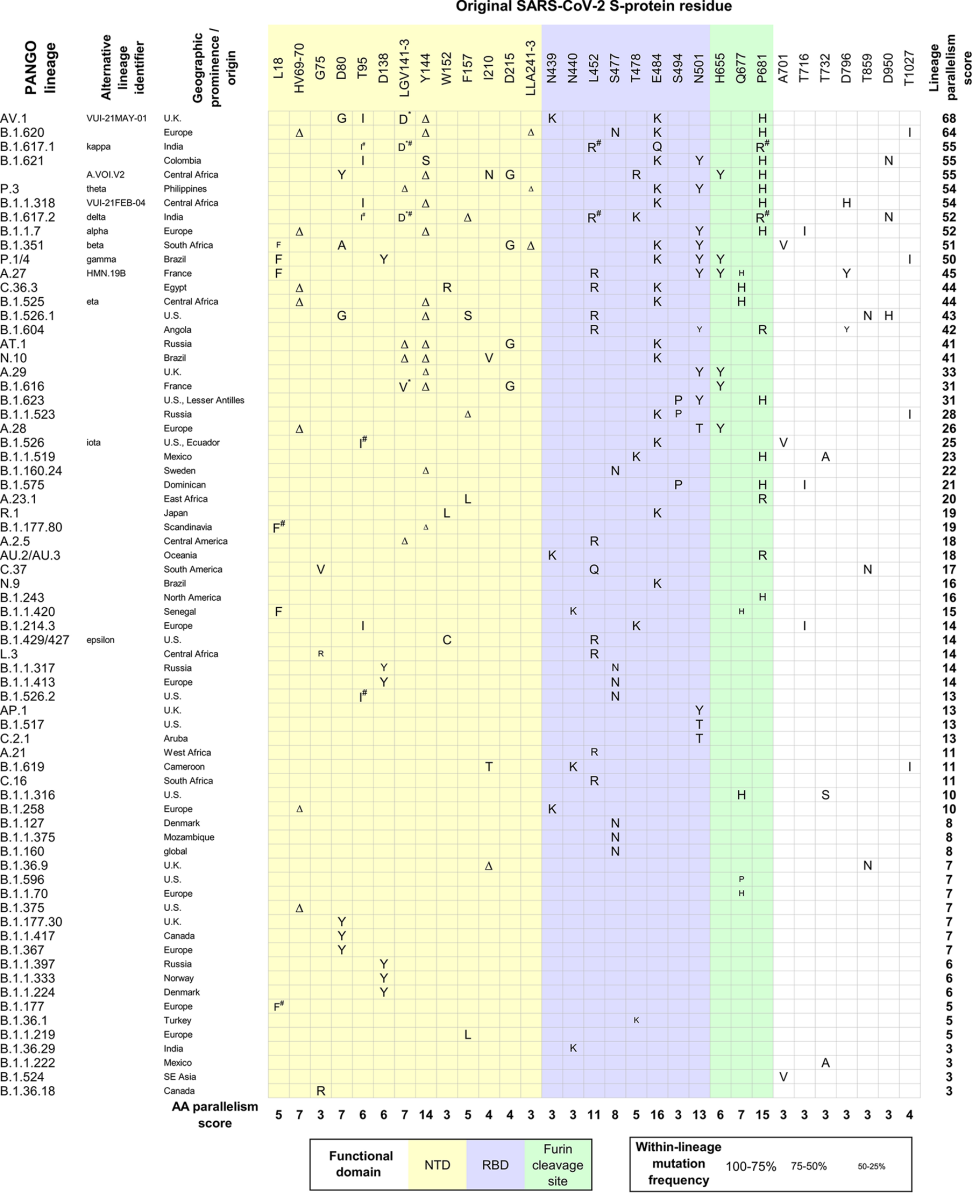
Convergent evolution of SARS-CoV-2 S-protein AA residues among different lineages (autumn 2020 – spring 2021). All S-protein AA mutations present in SARS-CoV-2 VOC lineages (alpha, beta, gamma, delta) were collected. The phylogenetic pattern of mutations at each of these AA positions was assessed visually in the representative global subsample of SARS-CoV-2 genomes (https://nextstrain.org/ncov/global) (Hadfield et al., 2018). NextStrain was scanned repeatedly between March to June 2021;lineages were confirmed to show a recent localized rise to high frequency (indicative of selective advantage). All additional S-protein AA mutations present in these lineages were subjected to the same procedure as above. After several iterations, a final set of S-protein positions that experienced ≥3 independent mutations was established. Information about the distribution and within-lineage frequency of S-protein mutations, and the spatiotemporal characterization of SARS-CoV-2 lineages was retrieved from (Tsueng et al., 2022) (15 June 2021). The AA parallelism score was established for all convergently mutated AAs, by summing their independent emergence events. The lineage parallelism score was calculated by summing the parallelism scores of S-protein AAs mutated in individual lineages. *AA substitutions for G142; Mutations inherited from the same progenitor are considered a single evolutionary origin in parallelism score calculations.

In the GISAID database (Shu and McCauley, 2017), only five percent of the S-protein amino acids (AAs) show mutations in more than 100,000 genomic sequences (out of 9 mil. genomic sequences in GISAID; March 15, 2022). By theory, the fitness advantage of a mutation translates into the increased representation of the viral lineage carrying it; the extreme example being a selective sweep/lineage replacement. This happened with the D614G mutation, which quickly became dominant during the early stages of the pandemic and which positive impact on the virus fitness is well recognized (Volz et al., 2021). However, most of the conspicuous mutations which keep rising in prevalence have emerged by convergent evolution and many of them are present in dominant delta and subsequently omicron lineages. The fact that the same mutations emerge independently in different viral lineages is a standalone, strong indicator that these changes confer an adaptive advantage towards the virus infectivity and easier spread in the population. Similarly, a fixation of multiple different amino acids with chemical similarities implicates the ongoing optimization at the given site. **Figure 1** shows the distribution of convergent S-protein AA mutations which emerged at least three times independently during the period of a dramatic SARS-CoV-2 evolution between autumn 2020 and spring 2021. This comparison identified a total of 31 S-protein sites under convergent evolution. As expected, the most concerning SARS-CoV-2 lineages carry the heaviest burden of convergent S-protein mutations (**Figure 1**). Since this analysis (purposefully) neglects mutations in other viral genes and their putative effects, it demonstrates that the S-protein mutations *per se* are strong determinants of the success of viral lineages hosting them, although rapid convergent evolution can be seen in other genes as well (De Maio et al., 2021; Majumdar and Niyogi, 2021).

### Convergently Evolved Mutations Cluster in Specific Locations in the Spike Protein

**Figure 2:**
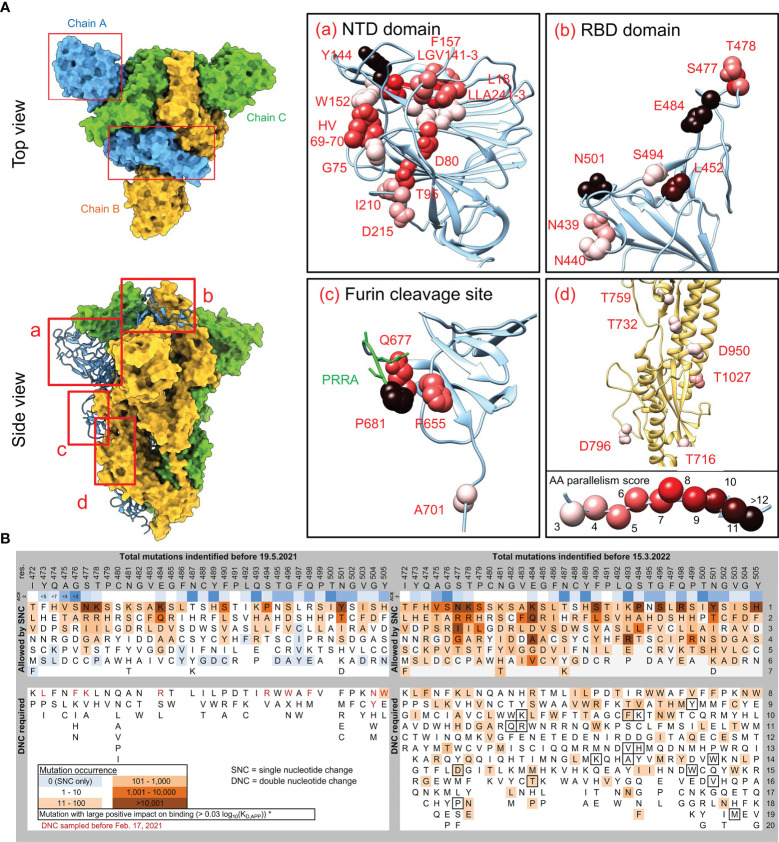
Localization of convergent mutations in SARS-CoV-2 S-protein structure, breakdown and progression of RBD AA mutations. **(A)** The S-protein parts missing in the crystal structure (PDB ID: 6zge) were modelled by the Modeller suite implemented in UCSF Chimera 1.13.1 (Webb and Sali, 2016). Residues under convergent evolution are depicted in spheres representation and colored according to their parallelism scores (**Figure 1**). Inset d) shows the region covering a portion of heptad repeat, central helix, and β-hairpin domains. Green residues in inset c) highlight the Furin cleavage site. **(B)** The mutational sequence space of RBD binding interface residues 472 – 505, and its coverage by mutations present in the GISAID database. All possible SNC (single-nucleotide change) AA substitutions are depicted. For TNC (two-nucleotide change) substitutions, only the subset of AA substitutions that were sampled in GISAID are depicted (in background color scale according to the legend), together with substitutions with a positive binding impact (frame). The AA position is invariant to its later occurrence to highlight differences. Deep-mutational scanning ΔLog_10_(*K*_D, App_) values were extracted from https://jbloomlab.github.io/SARS-CoV-2-RBD_DMS/ (Starr et al., 2020). ACE2 lane depicts residue distances in Å from the ACE2 receptor.

Mapping the convergently evolved mutations, at the time of the highest SARS-CoV-2 strains variability (between autumn 2020 and spring 2021), on the S-protein structure shows that the AAs under the strongest parallelism concentrate in three hotspots: N-terminal domain (NTD), receptor-binding domain (RBD) and the Furin cleavage site (**Figure 2A**). Such conspicuous nonrandom distribution pattern reflects their putative functional connection. All the most concerning variants carry one or (typically) more mutations in each of these three domains. The NTD domain accumulates the highest number of convergent mutations. Strikingly, all these AA residues, despite being located apart from each other in the S-protein primary structure, form perfectly co-localized patches, literally delineating the site of evolutionary pressure for optimization. We speculate that this adaption goes beyond a simple immune evasion or antigenic minimalization (Venkatakrishnan et al., 2021), which is unlikely to explain this level of convergence in NTD evolution. Instead, we suppose that NTD mutations might modulate some functional properties of this domain, which unfortunately remains experimentally understudied. The described NTD functions include controlling S-protein conformation (Li et al., 2021), interaction with host surface sialosides (Awasthi et al., 2020) and, most recently, binding to alternative entry receptors (Zhu et al., 2021). The second hot spot – the RBD domain shows a strong accumulation of mutations at the binding interface with the ACE2 receptor (see below for a dedicated paragraph). The function of the Furin cleavage site is well established (Xia et al., 2020; Johnson et al., 2021) as well as the relevance of the convergent Q677H mutation (Hodcroft et al., 2021). The impact of additional mutations in this region remains to be analyzed, but their positive fitness effect due to faster S-protein processing can be expected. As with the NTD, the Furin cleavage site AAs under convergent evolution are in direct contact with each other. The only convergent mutations not within these three domains are above residue 700, and their parallelization score is low (**Figure 2A**, inset d). It should be noted that these convergent mutations evolved on the background of a mostly naïve host. Conversely, the search for previously unseen convergent evolution in delta and omicron variants diversity since mid-2021 gained only three new significant mutations. A222V appeared at least three times among delta lineages, T95I emerged multiple times in delta and is present in omicron, and finally R346K was detected in omicron lineages.

### Combinations of Convergent Mutations Suggest Epistatic Relations

The spatial clustering of AAs under convergent evolution creates room for synergic effects between mutations, as was observed for the couple E484K/N501Y and proposed for Q498R/N501Y (Zahradník et al., 2021) that later emerged in omicron lineages. In addition, potential inter-domain relations in mutational appearance can be tentatively inferred. For example, as a whole, NTD convergent mutations predominate in lineages with mutated RBD. Furthermore, some NTD convergent mutations (e.g. T95I, ΔY144, D215G) accumulate in lineages with high parallelism scores and are never present alone (**Figure 1**). This indicates that the S-protein adaptive mutations emerge in a sequential manner. The precise order of mutation appearance is difficult to analyze since the lineages of concern with a high mutational load typically appear “out of nowhere” without a documented intermediate state. For the SARS-CoV-2 research, this means that, ideally, S-protein mutations should be experimentally evaluated in (both intra- and inter-domain) combinations.

### Exploring Double Nucleotide Changes by SARS-CoV-2 RBD

The RBD domain is the focus of our understanding of SARS-CoV-2 cell entry and neutralizing antibody evasion (Starr et al., 2020; Dejnirattisai et al., 2022), and its evolution thus deserves special attention. We generated an *in silico* library of all RBD AA mutations which can be achieved with a single nucleotide change (SNC) and two-nucleotide changes (TNC) of the original codons, and analyzed their global occurrence. Rapid progression can be seen as all of the possible SNC-dependent changes had been detected in early 2021 among the >1.2 million genomic sequences in GISAID. As the likelihood of two successive nucleotide changes of a single codon is much lower, AA mutations resulting from TNC appear among sampled genomes at much lower frequencies, yet their repertoire and numbers are growing over time and soon are likely to reach complete exhaustion (**Figure 2B**). The first TNC detected among VOC/VOI variants is omicron´s S371L mutation, with others predicted to follow. Some of the changes conferred by TNC are predicted to display a much tighter binding to ACE2 (**Figure 2B**) and thus represent a potential epidemiological risk. We can assume that the occurrence of epistatic mutations is also expanding, as seen for Q498R in the omicron. Taken together, the potential sequence space to be sampled in the future by the RBD to achieve an evolutionary advantage remains large and we will see a rise of previously unseen mutations.

### Both Receptor Binding and Immune Evasion Are Shaping the RBD Evolution

Most of the AA changes predicted to have a positive effect on ACE2 receptor binding [Log10(*K*_D,app_) > 0.03] (Starr et al., 2020) are present at significant frequencies among GISAD genomes (**Figure 2B**), reinforcing improved receptor binding as an importing contributor to the adaptive fitness gain. Mutations into positively charged residues are favored by electro-complementarity between RBD and ACE2 (Zahradník et al., 2021). Among the convergent RBD mutations, this concerns N439K, N440K, L452R, T478R/K and E484K (**Figure 1**). Expectedly, the selection of most mutations that negatively affect receptor binding is disfavored in RBD evolution (**Figure 2B**). Our predictions of SARS-CoV-2 evolution often involve immunocompromised patients, treated or non-treated with convalescent plasma or neutralizing antibodies (Tarhini et al., 2021; Truong et al., 2021), yet it is not clear how these two phenomena (global vs. within-patient virus evolution/diversification) mirror each other. While several RBD mutations have been selected during prolonged infection within the same immunocompromised patient (Choi et al., 2020; Clark et al., 2021), others were not e.g. the combination of E484K/N501Y, which increases the binding affinity to ACE2 receptor (Zahradník et al., 2021). In contrast, the NTD deletion Δ141-143 has evolved in multiple patients (Clark et al., 2021; Kemp et al., 2021) and, like ΔY144 (Chen et al., 2021), was clearly selected in response to the host environment. Some RBD mutations may increase viral fitness by immune evasion mechanisms other than reduced antibody neutralization, e.g. reduced MHC presentation [L452R (Motozono et al., 2021)]. It is thus evident that every mutation contributes to the viral fitness by different parameters and mechanisms, which need to be evaluated systematically.

### Global Selective Sweep Since Early 2021

The global evolutionary dynamics of SARS-CoV-2 has changed fundamentally since early 2021. The early common pattern (competition of multiple independent lineages on the background of the naïve population) gave way to the global selective sweep of delta VOC. Concomitantly, the rapid vaccination rate in combination with post-infection immunity made the virus face a significantly more immunized population. The second half of 2021 was characterized by the delta diversification into numerous competing sublineages. Finally, in November, omicron emerged from an unknown, presumably “near” wildtype SARS-CoV-2 (B.1.1) and has been globally spreading at an unprecedented rate. Omicron carries total 13 mutations in S-protein which were convergent in the pre-omicron era (HV69-70Δ, T95I, G142D, Y144Δ, N440K, S477N, T478K, E484A, N501Y, H655Y, P681H, D796Y, D950N), double of the maximum convergent mutations observed in any other VOCs so far. In this sense, omicron is an excellent example how these residues were built up again to form omicron, as no other variant had over seven of them (**Figure 1**) . In addition, the breakdown of RBD mutations prior of the omicron era showed omicron residues to be among the most prominent ones. The only exception is the mutation Q498R due to the already mentioned cooperativity to N501Y. Our work thus shows that SARS-CoV-2 early evolution was towards human adaptation which, despite remaining closed and unrepeatable, presaged future developments.

The extent and progression of convergence in S-protein evolution tentatively hints at the possibility that it might approach a final state of optimization to the novel, human host (Burioni and Topol, 2021). However, with the large mutation space available by rarer TNC and epistatic mutations, this is far from certain. Still, with the predictability and repeatability of S-protein evolution taken into consideration in vaccine design (even in omicron), the protection for the global population from the landscape of viral variants of current and near-future significance can be maximized. Clearly, mass vaccination and/or previous infections are altering the selective pressure and thus reshaping viral evolution. This phenomenon is already playing a role at position 484, where lysine is the optimal residue, but omicron variants is characteristic with E484A. It should be noted that omicron emerged in South Africa regions previously affected by E484K-bearing variants (gamma and beta). Also in omicron, the highly increased binding affinity of the Q498R/N501Y double-mutant was used to insert a slew of other mutations, that while reducing binding affinity promoted immune evasion (Dejnirattisai et al., 2022). In summary, taking S-protein convergent evolution into consideration could have provided us with the much-needed time to design and test broad-range VOC-effective vaccines in advance of the real developments. The need for global monitoring of SARS-CoV-2 evolution will, for some time, certainly remain a top priority.

## Data Availability Statement

Publicly available datasets were analyzed in this study. This data can be found here: GISAID.

## Author Contributions

JZ and GS conceived the project; JZ, JN, and GS performed experiments; JZ, JN, and GS wrote the manuscript. All authors contributed to the article and approved the submitted version.

## Funding

This research was supported by the Israel Science Foundation (grant No. 3814/19) within the KillCorona – Curbing Coronavirus Research Program and by the Ben B. and Joyce E. Eisenberg Foundation. JN acknowledges support by project MICOBION (H2020 No 81022), funded by Research Executive Agency (REA), and by the European Regional Development Fund and the Ministry of Education, Youth and Sports of the Czech Republic, grant number CZ.02.1.01/0.0/0.0/16_019/0000785.

## Conflict of Interest

The authors declare that the research was conducted in the absence of any commercial or financial relationships that could be construed as a potential conflict of interest.

## Publisher’s Note

All claims expressed in this article are solely those of the authors and do not necessarily represent those of their affiliated organizations, or those of the publisher, the editors and the reviewers. Any product that may be evaluated in this article, or claim that may be made by its manufacturer, is not guaranteed or endorsed by the publisher.
